# Diagnostic and Therapeutic Challenges in Parathyroid Cancers: 15 Years’ Experience in a Tertiary Center and an Endocrine Surgery Referral Hospital

**DOI:** 10.3390/jcm14061932

**Published:** 2025-03-13

**Authors:** Razvan Simescu, Andra Piciu, Valentin Muntean, Alexandru Mester, Doina Piciu

**Affiliations:** 1 Medlife-Humanitas Hospital Cluj-Napoca, 400664 Cluj-Napoca, Romaniavalentin.muntean@gmail.com (V.M.); 2 Department of Surgery, University of Medicine and Pharmacy “Iuliu Hatieganu”, 400012 Cluj-Napoca, Romania; 3 Department of Medical Oncology, University of Medicine and Pharmacy “Iuliu Hatieganu”, 400012 Cluj-Napoca, Romania; 4 Department of Oral Health, University of Medicine and Pharmacy “Iuliu Hatieganu”, 400012 Cluj-Napoca, Romania; 5 Doctoral School, University of Medicine and Pharmacy “Iuliu Hatieganu”, 400012 Cluj-Napoca, Romania; doina.piciu@umfcluj.ro; 6 Institute of Oncology Prof. Dr. I. Chiricuta Cluj-Napoca, Romania Center of Reference for Rare Endocrine Diseases—ENDO-ERN (European Reference Network on Rare Endocrine Conditions), 400015 Cluj-Napoca, Romania; 7 Affidea Cluj-Napoca, 400487 Cluj-Napoca, Romania

**Keywords:** parathyroid cancer, synchronous malignancies, immunohistochemistry

## Abstract

**Background:** Parathyroid cancers are rare endocrine malignancies that pose diagnostic and therapeutic challenges, particularly when discovered incidentally or in the presence of multiple endocrine disorders. This study aims to provide clinical, biochemical and pathological insights into these malignancies through a retrospective case series. **Methods:** We analyzed retrospectively, from a tertiary and an endocrine surgery referral center, 13 cases of parathyroid cancers, where 4 cases were associated with thyroid cancers, including demographic data, clinical presentation, biochemical markers, imaging, surgical interventions, histopathological findings and follow-up outcomes. Descriptive statistics were used to summarize patient characteristics. **Results:** The median age of the cohort was 64 (range: 40–81 years), with a female-to-male ratio of 8:5. More than half of the cases (61.53%) were diagnosed incidentally, with common biochemical findings including elevated parathyroid hormone (PTH) levels (median: 430 pg/mL) and hypercalcemia in 80% of the patients. All patients underwent surgery, with parathyroid resections with concomitant total thyroidectomy (62%) or lobectomy (23%) as the most common interventions. Histopathological analysis confirmed parathyroid carcinoma in all cases, with coexisting thyroid malignancies observed in 31%. An immunohistochemical profile performed in about half of the patients was in accordance with previously published data. Postoperative normalization of PTH levels was achieved in 77% of patients, and no recurrence or metastasis was observed in 85% of cases during follow-up. **Conclusions:** Despite the exceptional rarity of the disease, this case series highlights the importance of preoperative biochemical and imaging evaluation and the efficacy of surgical management. Long-term outcomes remain favorable with early diagnosis and diligent postoperative monitoring. Further research into molecular biomarkers and targeted therapies is warranted to improve the management of advanced or recurrent disease.

## 1. Introduction

Parathyroid cancers, though relatively rare, represent clinically significant endocrine malignancies with challenging diagnostics and therapies. Parathyroid carcinoma is one of the rarest malignant tumors, rating approximately 0.005%, with an incidence of 0.5–5% of all primary hyperparathyroidism (PHPT) cases [1,2]. It is associated with significant morbidity due to hypercalcemia and its complications, presenting unique diagnostic and therapeutic challenges [3,4,5]. In parallel, thyroid cancer, the most common endocrine malignancy, accounts for approximately 3% of all new cancer diagnoses globally, with its incidence continuing to rise over the past decades due to advances in diagnostic imaging and increased awareness [6,7]. Among its types, differentiated thyroid carcinoma (DTC), specifically papillary thyroid carcinoma (PTC), constitutes the majority of the cases, characterized by an excellent prognosis, while medullary (MTC) and anaplastic thyroid carcinoma (ATC) remain less frequent, but with more complicated natural history, either occurring in multiple endocrine neoplasia (MTC) and familial aggregation or having a highly aggressive evolution (ATC) [8]. Moreover, thyroid and parathyroid carcinoma is an exceptionally rare endocrine malignant association [9].

Advances in genomics and molecular biology have significantly enhanced our understanding of the pathophysiology of thyroid and parathyroid cancers. Mutations in the BRAF, RAS, and RET genes are well documented in thyroid cancers and have become pivotal in refining diagnostic algorithms and targeted therapies [10]. Parathyroid carcinoma is increasingly linked to defects in the CDC73 gene and alterations in the tumor suppressor protein parafibromin, underscoring the importance of molecular insights in disease management [11,12]. There is no standardized TNM staging system of parathyroid carcinoma, although proposals have been made [13,14]. Despite these advances, challenges persist in early diagnosis, risk stratification, and the development of effective treatments, particularly for aggressive forms.

Because of the rarity of parathyroid carcinomas, there are no international guidelines, neither for diagnostics nor for therapy, and even less for the association of both endocrine malignancies, which makes the lack of standardization pose significant pressure on patient and medical teams. In light of this evidence, every experience should be documented and communicated in order to offer knowledge for better care for our patients.

This article aims to provide the experience of a tertiary cancer center that has experienced more than 30 years in the field of endocrine tumors, combined with the expertise and knowledge of an endocrine surgery referral center team. By integrating current knowledge, we seek to address gaps in understanding and identify future directions for the management of these complex endocrine malignancies.

## 2. Materials and Methods

### 2.1. Study Design

This retrospective case series study analyzed demographic, clinical, biochemical, imagistic, pathological and outcome data of patients diagnosed with parathyroid cancers. The data were collected from the Institute of Oncology Prof. Dr. “Ion Chiricuta” Cluj-Napoca, Romania between January 2009 and December 2023 and from “MedLife-Humanitas” Endocrine Surgery Center Cluj-Napoca, Romania between June 2019 and December 2023. Ethics approval for this study was obtained from the Institutional Review Boards of the two hospitals involved and from the Ethics Committee of the University of Medicine and Pharmacy “Iuliu Hatieganu” Cluj-Napoca, Romania (Approval No. AVZ.30/20.02.2024). Patient consent was waived due to the retrospective nature of this study.

### 2.2. Study Population

Patients included in this study met the following criteria: diagnosed with parathyroid cancer, confirmed by histopathology; complete clinical, biochemical and follow-up data available in medical records; those who underwent surgical intervention with or without adjuvant therapy. This study also included patients with associated thyroid cancers confirmed in histopathology. Patients with incomplete data or diagnoses unrelated to thyroid or parathyroid cancers were excluded. Demographic, clinical and pathological data were extracted from medical records.

### 2.3. Biochemical and Imaging Evaluation

Preoperative levels of serum parathyroid hormone (PTH), corrected calcium, thyroid function tests (thyroid stimulated hormone-TSH, free thyroxine-FT4) and tumor markers such as thyroglobulin (Tg), anti-thyroglobulin, carcinoembryonic antigen (CEA) or calcitonin (Ct) were documented.

Imaging modalities such as ultrasound, computed tomography (CT) and positron emission tomography–computed tomography (PET-CT) were used to assess tumor characteristics and metastases. Ultrasound (US) of the neck was performed in all patients using different equipment over the years. CT of the neck, MRI and PET-CT with F18-fluorodeoxyglucose (F18-FDG) performed on PET/CT GE Optima were indicated in some cases to evaluate staging or local invasion. For diagnostic reasons, fine-needle aspiration (FNA) on parathyroid glands and thyroid nodules was performed in a limited number. Tc-99m scintigraphy with MIBI (Tc-99m methoxy–isobutyl–isonitrile) (planar or SPECT/CT) was performed in some cases in order to localize the affected parathyroid gland, using Siemens E-cam Signature gamma camera or GE Optima 560 camera, in two standard protocols: dual tracers (Tc-99m pertechnetate/Tc-99m MIBI) or a single washout tracer (Tc-99m MIBI early and delay images), using an LEHR collimator, 256x256 matrix and three incidences (anterior–posterior, latero-lateral, oblique–lateral) of the neck and upper mediastinum, according to the EANM guidelines [15]. Where indicated, PET-CT with F18-fluorodeoxyglucose (F18-FDG) was performed on PET/CT GE Optima 560.

### 2.4. Histopathological and Immunohistochemical Analysis

Histopathological examination of surgical specimens was performed according to standard protocols of the specific staining of hematoxylin–eosin and the PTH marker.

One of the following microscopic features is necessary for a definitive diagnosis of a parathyroid lesion malignancy: angioinvasion (vascular invasion), lymphatic invasion, perineural (intraneural) invasion, invasion of adjacent structures/organs [16] or metastasis (lymph node or distant) biopsy confirmation [17]. Immunohistochemistry (IHC) was used to evaluate specific markers (TTF1, CK19, CD34, CD31, cyclin D1, blc2 and Ki-67 proliferation index), aiding in the differential diagnosis of thyroid and parathyroid malignancies. In about half of the cases, histopathology was reviewed by a second pathologist who examined the hematoxylin–eosin slides and performed IHC analysis of the paraffin-embedded tissue sections for the parafibromin, galectin-3, E-cadherin, cyclin D1 and Ki-67 proliferation index on an automated IHC system (Agilent-Dako Autostainer Link 48) (Table 1).

### 2.5. Follow-Up and Outcome Measures

Follow-up data included postoperative PTH levels, imaging results and recurrence or metastasis. Disease-free survival (DFS) and overall survival (OS) were recorded from the date of surgery to the last follow-up or the occurrence of events.

### 2.6. Statistical Analysis

Categorical variables were presented as frequencies and percentages, while continuous variables were expressed as mean ± standard deviation (SD). Normality was assessed using the Shapiro–Wilk test. Group comparisons were performed using Student’s *t*-test/Mann–Whitney U test for continuous variables and Chi-square/Fisher’s exact test for categorical data. Kaplan–Meier analysis with the log-rank test was used for survival analysis, and a Cox proportional hazards model evaluated prognostic factors. A *p*-value < 0.05 was considered as statistically significant.

## 3. Results

### 3.1. Study Population

This retrospective case series included 13 patients diagnosed with parathyroid cancers with, among them, 4 patients presenting synchronous thyroid and parathyroid cancers. The median age was 64 years (range: 40–81 years), the mean age of the patients was 61.84 ± 10.55 years and females had a slightly higher median age (67 years) compared to males (57 years). Females represented 62% (eight patients) of the cohort while males accounted for 38% (five patients). More than half of the diagnoses (61.53%, eight patients) were incidental, identified during imaging for unrelated conditions or routine screenings.

None of the patients had an irradiation history, nor any relevant family history, including genetic syndrome evidence.

### 3.2. Clinical and Biochemical Findings

In three patients, PTH and calcium were not evaluated before their operations. Of the other patients, eight patients (80%) had elevated preoperative PTH levels, with a median of 430 pg/mL (range: 54–1992 pg/mL) and mean of 707.1 ± 635.02 pg/mL. A PTH level five times higher than the upper normal value was seen in 70% of the patients. Hypercalcemia was documented in seven patients, with serum calcium levels ranging from 9.1 to 15.2 mg/dL, with a median of 14.1 mg/dL and mean of 13.15 ± 1.96 mg/dL. Half of the patients exhibited values over 14 mg/dl. Common signs and symptoms associated with hyperparathyroidism included joint pain, renal lithiasis and osteoporosis, which were reported in 46% of cases. A total of 83% of these patients had at least three systems affected by the disease. One patient presented a palpable solid nodule, which proved to be the parathyroid gland. One case of parathyroid carcinoma was a non-secreting PC, and another showed hypercalcemic crisis. Regarding the thyroid function, 77% of the patients had normal hormonal secretion, two had hypothyroidism due to Hashimoto’s thyroiditis and one had hyperthyroidism. Calcitonin and CEA levels were evaluated in three patients, one having nonspecific elevated Ct, which was elucidated by the histopathology finding of C-cell hyperplasia (Table 2).

### 3.3. Imaging Findings

All patients were examined preoperatively by neck ultrasound. Parathyroid lesions were identified in 62% of these. In seven of the eight cases (87.5%), suspicious features for PC (diameter > 3 cm, ill-defined borders, cystic component or calcifications) were identified. In one case, a CT scan showed a suspicious tumor of unknown origin apparently adherent to the thyroid gland. It finally proved to be the non-secreting PC. Localization of the affected parathyroid gland was completed by Tc-99m scintigraphy with MIBI (planar or SPECT/CT) in three, cases with positive concordance to US; no supernumerary or ectopic parathyroid glands were shown. In two patients, brown tumors were diagnosed by CT and DEXA scans. A thyroid nodular pathology was diagnosed in seven (62%) patients, with suspicious features (hypoechoic, ill-defined borders or microcalcifications) identified in four of the seven cases (57.14%). Two of these cases had fine-needle aspiration cytology (FNAC) with Bethesda IV and V reports.

### 3.4. Surgical Interventions

All 13 patients underwent surgical intervention. The most frequent procedure was bilateral parathyroid exploration with parathyroidectomy and concomitant total thyroidectomy, performed in 62% of cases (eight patients). In two cases, selective lymph node excision was associated. Lobectomy with parathyroidectomy was performed in 23% of cases (three patients). Intra- or immediate postoperative PTH levels showed a significant reduction (>50% drop) in all patients and normalization in 70% of cases (nine patients). The median postoperative PTH level was 54.1 pg/mL (range: 5.8–175 pg/mL) and the mean was 67.32 ± 48.72 pg/mL.

### 3.5. Histopathological Findings

Parathyroid carcinoma was confirmed in all 13 cases, with varying degrees of invasion. Coexisting thyroid conditions were observed in four cases: classic papillary thyroid carcinoma in three patients and follicular–papillary thyroid cancer and Hashimoto’s thyroiditis in one patient.

Immunohistochemistry revealed tumor marker patterns characteristic of malignancy, including parafibromin and E-cadherin loss and positivity for markers such as galectin-3 and cyclin D1 in parathyroid cancers and TTF1 and CK19 in thyroid cancer. In three out of nine (33.33%) patients with Ki67, the proliferative index was determined to be over 5%, and three more patients exhibited values of 5. The median Ki67 value was 5% (range: 1–7%) and the mean was 3.79 +/− SD 2.30%. Parafibromin and E-cadherin staining was negative in each of two (33.33%) of the six analyzed cases. Two more patients had weak parafibromin staining. Galectin-3 was positive in two (33.33%) patients as well. Of note, one PC was parafibromin-negative and galectin-3-positive. Cyclin D1 was positive in the two cases where it was used by the pathologist (Table 3 and Table 4).

### 3.6. Adjuvant Therapies

In three cases, radioiodine (I-131) therapy was performed with the following single-dose activities: 1.85 GBq, 2.56 GBq and 3.7GBq of I-131 (Table 5). In the case of non-secreting parathyroid carcinoma with muscle invasion, four cycles of chemotherapy with paclitaxel and 40 Gy of external beam radiotherapy were administered.

### 3.7. Outcomes

Over a mean follow-up duration of 41 months (range: 12–180 months), 11 patients showed no evidence of recurrence or metastasis. One patient exhibited a pulmonary nodule seen on CT and F18-FDG PET-CT. The case was associated with thyroid cancer and, on the whole-body scan with I-131 (WBS I-131), the pulmonary nodules showed no uptake, underlying the parathyroid origin, also confirmed on biopsy. In a second case, thoracic CT scans revealed suspicious non-FDG-avid pulmonary nodules on consequent PET-CT scans. No disease-related mortality or significant disease progression was reported during follow-up, but 31% of patients had persistent slightly elevated PTH values, ranging between 85 and 115 pg/mL.

## 4. Discussion

This case series provides valuable insights into the clinical, biochemical and histopathological characteristics of thyroid and parathyroid cancers. Although these malignancies are rare, they pose significant diagnostic and therapeutic challenges, especially in cases where they are discovered incidentally or coexist with other endocrine disorders.

Our cohort of patients, although low in number, had a similar epidemiological profile to what was previously published: the female-to-male ratio does not show an over-2:1 distribution in favor of female patients as seen in non-malignant PHPT, and is comparable to other studies [14,18,19,20]. Also, the median age at presentation of 64 years, although high, is within the 44–65 years range identified by an extensive review published in recent years [13].

The predominance of incidental diagnoses (61.53%) in this study underscores the importance of routine imaging and biochemical assessment in detecting thyroid and parathyroid pathology. Incidental parathyroid carcinoma remains a well-documented phenomenon, often presenting asymptomatically or with subtle hypercalcemia and elevated parathyroid hormone (PTH) levels. Studies indicate that nearly 20–50% of parathyroid carcinomas are discovered during evaluation for hyperparathyroidism, emphasizing the need for heightened clinical vigilance in such cases [21].

Hyperparathyroidism-related complications, including joint pain, osteoporosis and renal lithiasis, were present in 46% of the patients in this cohort, mirroring findings in the literature that associate these symptoms with prolonged, indolent or advanced disease [1]. Of these patients, 80% had at least three systems affected by the disease, and all of them had simultaneous bone and kidney involvement. High suspicion for PC was suggested if both renal and bone signs or symptoms are present in a patient with hyperparathyroidism, as this simultaneous occurrence is quite rare in benign cases [22].

Previous studies have suggested that PC could be suspected in patients with severe hypercalcemia (>14 mg/dL), a big size of the tumor and/or with marked elevated PTH levels (>5 times the upper normal limit) [2,23]. This is seen in our small cohort as well, in which 70% of the patients had marked elevated PTH levels and 50% had significant hypercalcemia. To date, approximately 30 cases of non-secreting PC were reported, the majority posing serious diagnostic challenges, including our one case, which was extensively detailed in a previous case report [24].

Careful cervical US is mandatory before any operation on the thyroid or parathyroids, with focus on all structures regardless of surgical indication. Previous studies of preoperative US identified several PC suspicious features: a size > 3 cm, heterogeneous structure, evidence of degeneration (cystic inclusions, calcifications) and lobulated, irregular or ill-defined borders [25,26,27]. Also, the neck ultrasound is highly sensitive for the detection of thyroid carcinoma, especially PTC [28,29,30]. In our cohort, preoperative US suspicions features were identified in 87.5% (seven out of eight patients) of the PCs and in 75% (three out of four patients) of the PTCs. These findings, together with our previous review on the association of PC and differentiated thyroid carcinomas, suggest that neck US has the greatest potential to raise both PC and PTC suspicion [9].

The significant reduction in postoperative PTH levels in 77% of patients highlights the efficacy of surgical intervention, especially when concomitant or en bloc resection of PC and the thyroid is performed (84% in our cohort), which remains the cornerstone of treatment for parathyroid carcinoma. The normalization of PTH levels postoperatively is strongly associated with improved biochemical outcomes and symptom resolution [31].

Parathyroid carcinoma was associated with a minority of patients also diagnosed with thyroid carcinoma: 33 patients were identified in publications until 2022 [9] and 4 more cases (31%) were identified in the present study. This association underscores the importance of thorough preoperative evaluation, including imaging and fine-needle aspiration, to identify multifocal or synchronous disease.

The role of immunohistochemistry in diagnosis was critical, with markers such as Ki67%, parafibromin, galectin-3, TTF1 and CK19 aiding in differentiating benign from malignant lesions. For the multitude of IHC markers tested, negative parafibromin and galectin-3, and also a Ki-67 proliferation index > 5%, have the strongest evidence base for PC diagnosis [32,33]. In our cohort, Ki67 > 5% was present in 20% of the analyzed specimens, which ranges within previously published percentages of 5.8–85% [33]. The loss of parafibromin and galectin-3 expression has been linked with tumor aggressiveness and recurrence risk [18,34,35]. In our IHC study, the one patient with confirmed pulmonary metastasis had both parafibromin- and galectin-3-negative staining on the primary tumor.

In terms of prognosis, our findings align with prior studies, demonstrating favorable survival outcomes for patients with early-stage parathyroid carcinoma, as 11 of the 13 patients in our study had no recurrence or metastasis during a median follow-up of 41 months. However, persistent pulmonary nodules in two patients highlight the need for continued monitoring. Previous research has shown that long-term recurrence rates for parathyroid carcinoma can reach up to 30%, even in surgically treated patients, necessitating regular imaging and biochemical follow-up [19,36,37].

## 5. Conclusions

This study contributes to the growing body of evidence on the clinical and pathological characteristics of parathyroid cancers. While surgical resection remains highly effective, long-term outcomes depend on early diagnosis, comprehensive preoperative assessment and diligent postoperative surveillance. Future research should focus on molecular biomarkers and targeted therapies to improve prognosis for advanced or recurrent disease.

## Figures and Tables

**Table 1:** Characteristics of antibodies used in IHC analysis.

Antibody	Cellular Location	Species/Antibody Specificity	Source	Clone No.	Dilution
Ki67	Nuclear	Mouse/monoclonal	Dako	MIB-1	RTU
Parafibromin	Nuclear	Mouse/monoclonal	BioSB	BSB-50	1:60
Galectin-3	Nuclear	Mouse/monoclonal	BioSB	9C4	1:100
E-cadherin	Cytoplasmatic/Extracelullar	Mouse/monoclonal	Dako	NCH-38	RTU
Cyclin D1	Nuclear	Rabbit/monoclonal	Dako	EP12	RTU

**Table 2:** Epidemiological and biochemical characteristics and surgery details of patients with parathyroid cancers.

Pts No.	1	2	3	4	5	6	7	8	9	10	11	12	13
Age (yo)	67	64	81	48	54	62	67	72	57	54	70	40	68
Gender	F	F	M	M	F	M	F	F	M	M	F	F	F
Corrected calcium (mg/dL)	14.9	-	12.1	14.1	9.1	-	-	14.5	15.2	14.1	14.7	10.4	11.2
PTH (pg/mL)	267	-	390	778	54 (nonsecreting PC)	-	-	1992	470	1207	1413	127	373
Postop. PTH (pg/mL)	121	54.1	5.8	104	61.1	26.4	14.1	43.8	42	71.5	115	41.3	175
Type of operation	PE	PE + TT	PE + TT	PEbLTL + RTLR	PE + TT + SLNE	PE + TT	PE + TT	PEbRTL	PEbLTS	PEbRTL + LTSR	PEbRTL	PE	PEbRTL + LTSR + CLNE

F—female; M—male; PC—parathyroid carcinoma; PTH—parathormone; PE—parathyroid excision; BPE—bilateral parathyroid exploration; PEbLTL—parathyroid excision “en bloc” with the left thyroid lobe; PEbRTL—parathyroid excision “en bloc” with the right thyroid lobe; RTLR—right thyroid lobe resection; LTLR—left thyroid lobe resection; SLNE—selective lymph node excision; CLNE—central lymph node excision.

**Table 3:** Pathological characteristics, adjuvant treatment and follow-up data of patients with parathyroid cancers.

Features	No. of Patients (%)
Capsular invasion	11 (84.62%)
Vascular invasion	5 (38.46%)
Soft tissue invasion	1 (7.69%)
Lymph node metastases	1 (7.69%)
Distant metastases	2 (15.38%)

**Table 4:** Immunohistochemistry profile in 6 cases of the cohort.

IHC	Case No. 8	Case No. 9	Case No. 10	Case No. 11	Case No. 2	Case No. 13
Ki67%	3	5	1.3	5	7	1.6
PFB	+	w+	w+	-	+	-
Gal-3	-	-	-	+	-	+
E-cad	+	-	+	+	+	+
CyclinD1	N/A	+	N/A	N/A	+	N/A

IHC—immunohistochemistry; PFB—parafibromin; Gal-3—galectin-3; E-cad—E-cadherin; “+”—positive stain; “-“—loss of expression; w+—weak positive stain; N/A—not available.

**Table 5:** Cases of parathyroid cancers and thyroid carcinoma.

ThyCC	Case No. 2—PC	Case No. 4—PC	Case No. 6—PC	Case No. 13—PC
Age (years)	64	48	62	68
Type	PTC	PTC	mPTC bilateral	PTC
Variant	Conventional	Conventional	Conventional	Follicular
TNM+	pT1bNxMx L0V0Pn0R0	pT2NxMx L1V0Pn0R0	pT1a(m)N0Mx L0V0Pn0R0	pT2N1aMx L1V0Pn0R0
Stage	I	I	I	II
Adj. I-131 therapy	1.85 GBq	2.56 GBq	-	3.7 GBb

ThyCC—thyroid cancer; PC—parathyroid carcinoma; PTC—papillary thyroid carcinoma; mPTC—micropapillary thyroid carcinoma; T—tumor; N—lymph node; L—lymphatic vessel invasion; V—vascular invasion; Pn—perineural invasion; R—resection margins. Adj—adjuvant.

## Data Availability

The data presented in this study are available on request from the corresponding authors.
